# The transcriptional regulator GalR self-assembles to form highly regular tubular structures

**DOI:** 10.1038/srep27672

**Published:** 2016-06-09

**Authors:** Emil D. Agerschou, Gunna Christiansen, Nicholas P. Schafer, Daniel Jhaf Madsen, Ditlev E. Brodersen, Szabolcs Semsey, Daniel E. Otzen

**Affiliations:** 1 Interdisciplinary Nanoscience Center (iNANO), Department of Molecular Biology and Genetics, Aarhus University, Gustav Wieds Vej 14, 8000 Aarhus C, Denmark; 2 Department of Biomedicine, Aarhus University, Wilhelm Meyers Allé 4, 8000 Aarhus C, Denmark; 3 Department of Molecular Biology and Genetics, Aarhus University, Gustav Wieds Vej 10c, DK-8000 Aarhus C, Denmark; 4 Department of Biology, University of Copenhagen, Ole Maaløesvej 5, 2200 København N, Denmark.

## Abstract

The Gal repressor regulates transport and metabolism of *D*-galactose in *Escherichia coli* and can mediate DNA loop formation by forming a bridge between adjacent or distant sites. GalR forms insoluble aggregates at lower salt concentrations *in vitro*, which can be solubilized at higher salt concentrations. Here, we investigate the assembly and disassembly of GalR aggregates. We find that a sharp transition from aggregates to soluble species occurs between 200 and 400 mM NaCl, incompatible with a simple salting-in effect. The aggregates are highly ordered rod-like structures, highlighting a remarkable ability for organized self-assembly. Mutant studies reveal that aggregation is dependent on two separate interfaces of GalR. The highly ordered structures dissociate to smaller aggregates in the presence of *D*-galactose. We propose that these self-assembled structures may constitute galactose-tolerant polymers for chromosome compaction in stationary phase cells, in effect linking self-assembly with regulatory function.

GalR is the main regulator of galactose transport and metabolism in *Escherichia coli*. The GalR protein can be purified as a homodimer of a 37 kDa subunit at 0.6 M KCl^1^. The dimeric repressor protein can bind to specific 16-bp operator sites and regulate transcription of the associated promoters. GalR represses transcription by diverse mechanisms including steric hindrance, contact inhibition, and DNA looping^2^,^3^,^4^,^5^. GalR can also activate transcription by interacting with the C-terminal domain of the α-subunit of RNA polymerase^6^. Repression of the *P1* and *P2* promoters of the *galETKM* operon by DNA looping requires assembly of a higher order nucleoprotein complex, the Gal repressosome. Repressosome formation requires (i) binding of two individual GalR dimers to two operator elements separated by 113 base pairs^2^,^7^,^8^; (ii) negatively supercoiled DNA^9^; (iii) optimal angular orientation of the two operator sites^10^; (iv) direct interaction of the two DNA-bound GalR dimers, looping out the intervening DNA segment that contains the *P1* and *P2* promoters^11^,^12^; and (v) specific binding of the HU protein to a DNA site (*hbs*) in the inter-operator region^13^. Increased inter-operator distance or enhanced GalR tetramerization alleviates the requirement of supercoiling and HU binding^12^,^14^. Structure-based genetic analysis has defined the GalR surfaces interacting to form a stacked, V-shaped, tetrameric structure^15^,^16^,^17^. Finally, binding of *D*-galactose (*D*-gal) to GalR inhibits both DNA binding and tetramerization^18^,^19^.

GalR can self-associate to the level of octamers that can connect two or more segments of DNA on small synthetic plasmids^20^ and to higher order insoluble structures^1^. An intriguing feature of GalR self-association is its regulation by salt. At less than 0.2 M salt, GalR forms aggregates that precipitate, a process which is at least partially reversed in the presence of 0.6 M KCl^1^. The observed reversibility suggests that the aggregation process could be a salt-dependent thermodynamic equilibrium between soluble and aggregated GalR. The structure of GalR in these aggregates remains unknown.

The low ionic strength in the protoplasm of cells grown in typical media (~150 mM) should favor GalR aggregation^21^. Indeed, a recent study where GalR was fused to the fluorescent Venus protein showed that GalR likely exists *in vivo* in an aggregated form when cells are in stationary phase^20^. Association of the DNA-bound GalR dimers was suggested both to allow coordination of regulation at distant promoter sites and to contribute to overall nucleoid architecture^20^. *In vivo* aggregation requires tetramerization of GalR, as almost no aggregates were formed when the Venus protein was fused to a non-tetramerizing GalR mutant^20^. Although tetramerization of GalR is inhibited *in vitro* in the presence of *D*-galactose^19^, the GalR foci formed in stationary phase cells persisted in the presence of *D*-galactose when cells were grown in minimal medium with fructose which does not interfere with induction of the *galETKM* operon^22^. These results suggest that the GalR aggregates represent structures of biological relevance and highlights the importance of understanding the structural determinants of their self-assembly.

In this paper we address the microscopic structure of the low-salt GalR aggregates, the conformational changes in GalR associated with aggregation, and the regulation of the assembly and disassembly of the aggregates. Together, our results reveal GalR’s ability to self-assemble to exquisitely detailed higher-order tubular structures on the scale of several hundred nm. We suggest that aggregation of GalR to these assemblies may allow a novel type of regulation.

## Results

### GalR forms highly ordered aggregates in a salt-dependent manner

**Figure 1:**
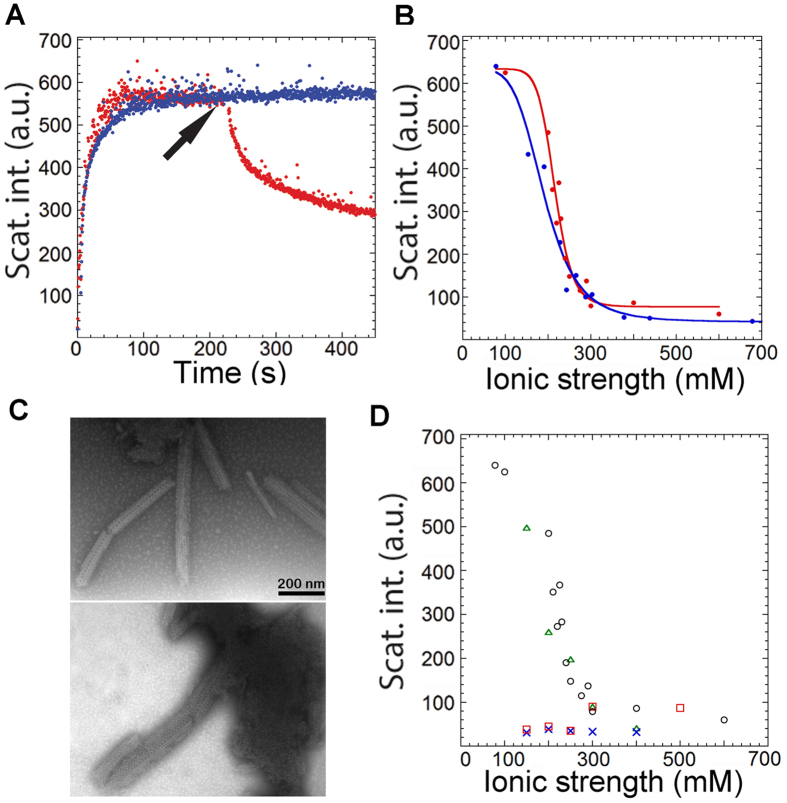
(**A**) Light scattering of 2 μM GalR^WT^ after dilution in a buffer containing a final concentration of 150 mM NaCl (blue curve). The arrowhead indicates addition of D-galactose (red curve). The scattering intensity is shown in arbitrary units (a.u.). (**B**) Plateau values of light scattering reached after the rapid phase of aggregation. 2 μM GalR^WT^ was diluted in buffers containing different [NaCl] (red dots) or [Na_2_SO_4_] (blue dots) and the plateau values were plotted against ionic strength. Lines are the fits to the sigmoid equation. R^2^ values are 0.972 (red) and 0.966 (blue). (**C**) Two different NS-TEM images of ordered aggregates formed after dilution of GalR^WT^ in a buffer containing 250 mM NaCl and incubation for 1 h. (**D**) Plateau values of light scattering of 2 μM GalR^WT^ (black), GalR^Y244F^ (green), GalR^T322R^ (blue), and GalR^Δ46^ (red) at different [NaCl], plotted against ionic strength.

During purification, GalR can be kept soluble using buffers containing 1 M NaCl, while reduction to less than ~200 mM leads to visible turbidity, even though the protein is diluted in the process of reducing the salt concentration^1^. We followed the aggregation process over time using light scattering at 365 nm, observing a rapid rise in scattering intensity which leveled off to a plateau over a few minutes (Fig. 1A). Addition of *D*-galactose led to a partial reduction in scattering (Fig. 1A), as well as a drop in absorbance (Fig. S1A), suggesting either that aggregated GalR retains sufficient native structure to be able to bind *D*-galactose and dissociate, or the aggregate is in dynamic equilibrium with native dimeric species which can bind galactose and thus displace the equilibrium towards the soluble state. Intrinsic Trp fluorescence spectra did not allow us to distinguish between the these two scenarios; they did not shift peak position but underwent a reduction in intensity, suggesting a change in the environment around Trp (Fig. S1B). The plateau level of scattering intensity scaled linearly with protein concentration and extrapolated to ~0 at zero protein concentration (Fig. S1C), implying to a first approximation that the same type of aggregates (with the same light scattering properties) were formed at all concentrations. However, the plateau intensity showed a sigmoidal dependence on [NaCl] (Fig. 1B). A similar type of behavior was observed in the presence of the divalent salt Na_2_SO_4_ (Fig. 1B). These two anions have very different salting-out capabilities, but the close overlap of these two data sets when plotted against ionic strength strongly suggests that aggregation is controlled by screening of electrostatic interactions. Centrifugation followed by analysis of supernatant and pellet by SDS-PAGE revealed that GalR was predominately found in the pellet fraction at lower [NaCl] and in the supernatant at high [NaCl] (Fig. S1D). Negative staining transmission electron microscopy (NS-TEM) confirmed that aggregates were increasingly formed at low salt, while few aggregates were observed at high salt. Remarkably, we observed highly ordered structures around 250 mM NaCl after 1 h incubation (Fig. 1C), corresponding to the transition region in Fig. 1B. Highly ordered aggregate structures were also observed at 400 mM NaCl when the protein concentration was 4-fold increased (Fig. S1E), indicating that higher protein concentrations can partially compensate for electrostatic screening. (Note that for simplicity we retain the term “aggregate” in the following description of GalR assembly as a general term for different types of association).

**Figure 2:**
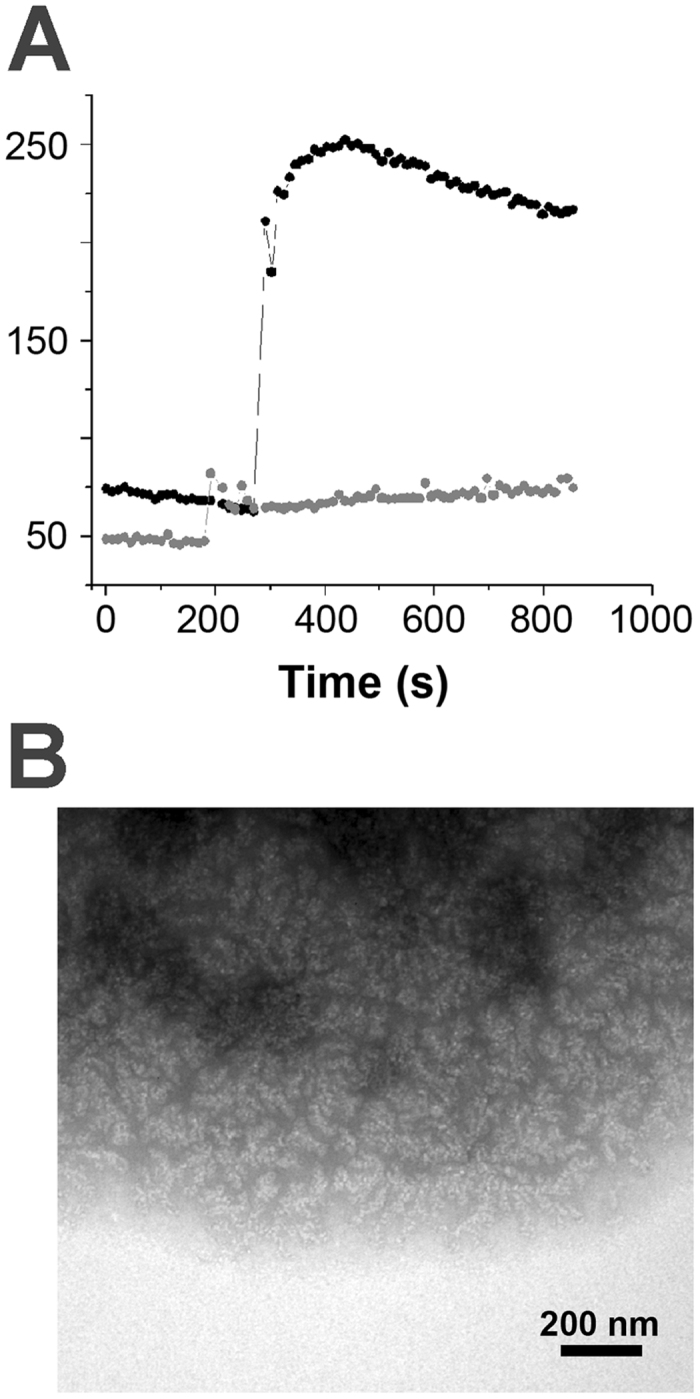
(**A**) Light scattering of 2 μM GalR^WT^ (black) and GalR^N48I^ (grey) after dilution in a buffer containing a final concentration of 150 mM NaCl. (**B**) NS-TEM images of aggregates formed after dilution of GalR^N48I^ in a buffer containing 150 mM NaCl. The initial ordered structures could be observed, but, unlike in the case of GalR^WT^ (Fig. 8A), the highly ordered polymers did not emerge from these structures.

To test how aggregation could be linked to the self-associative and ligand-binding properties of GalR, we studied the aggregation of four mutants using light scattering and NS-TEM. For GalR^T322R^, tetramer formation is strongly impaired, while GalR^Y244F^ is incapable of binding to *D*-galactose but can still tetramerize^23^. GalR^Δ46^ has a truncated N-terminus (*i.e.* does not have the DNA binding head piece), while GalR^N48I^ is defective in DNA binding^24^. GalR^T322R^ only aggregates to a very small degree *in vivo*^20^ and consistent with this, GalR^T322R^ shows a very low level of aggregation *in vitro* (Fig. 1D). In contrast, GalR^Y244F^ responded to changing [NaCl] in roughly the same way as GalR^WT^. GalR^Y244F^ aggregation was insensitive to *D*-galactose (Fig. S1A) but was more sensitive to [NaCl] than GalR^WT^, although the same types of highly ordered structures were observed (data not shown). GalR^Δ46^ showed the same low light scattering properties as the GalR^T322R^ mutant, although with a slight increase in scattering at high [NaCl]. GalR^N48I^ showed only a slight increase in scattering compared to GalR^WT^ after dilution of the protein in a buffer containing a final concentration of 150 mM NaCl (Fig. 2). Impaired aggregation of GalR^N48I^ correlated with the *in vivo* behavior of the protein. In cells carrying GalR^N48I^-EGFP fusions we observed uniform distribution of fluorescence, unlike in the case of GalR^WT^-EGFP, where fluorescent foci appeared as previously reported^20^ (Fig. S2).

The presence of supercoiled plasmid DNA containing GalR operators did not inhibit GalR^WT^ aggregation, and association of aggregates and the plasmid DNA was observed (Fig. S3). Furthermore, fluorescent foci appeared in cells carrying GalR^V7A^-EGFP fusions, which is unable to bind DNA^24^, suggesting that *in vivo* aggregation of GalR can occur in the absence of specific binding to GalR operators (Fig. S2).

Based on these observations we concluded that both the tetramerization domain and the headpiece containing the DNA-binding domain, but not the ability to bind *D*-galactose, are important for GalR aggregation. However, the ability of GalR to bind to DNA *per se* is neither required for nor interferes with aggregation (Figs S2 and S3).

### The kinetics of GalR aggregation and formation of structured polymers

**Figure 3:**
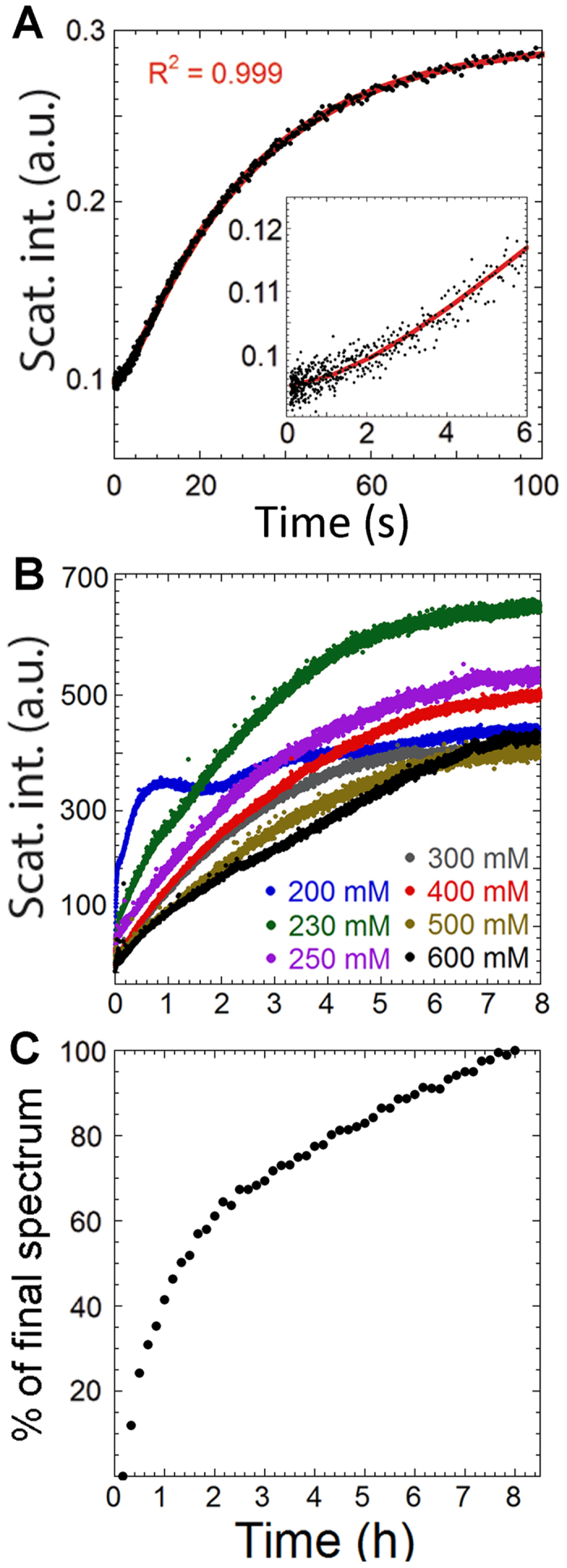
(**A**) Stopped-flow kinetics of 2 μM GalR^WT^ diluted in a buffer containing 150 mM NaCl (black dots). Red line represents the fit to Eq 1 with the corresponding R^2^ shown. Inset is a zoom in on the first 6 seconds. (**B**) Light scattering traces of 2 μM GalR^WT^ diluted in buffers containing different [NaCl] recorded for 8 hours. The appearance of the 200 mM trace was reproducible. (**C**) The time development of CD spectra at 250 mM NaCl (see also Fig. S5A,B) shown as the relative amount of the final spectra recorded, as obtained by least square method where all spectra in the series were described as a linear combination of a first and final spectra.

**Figure 4:**
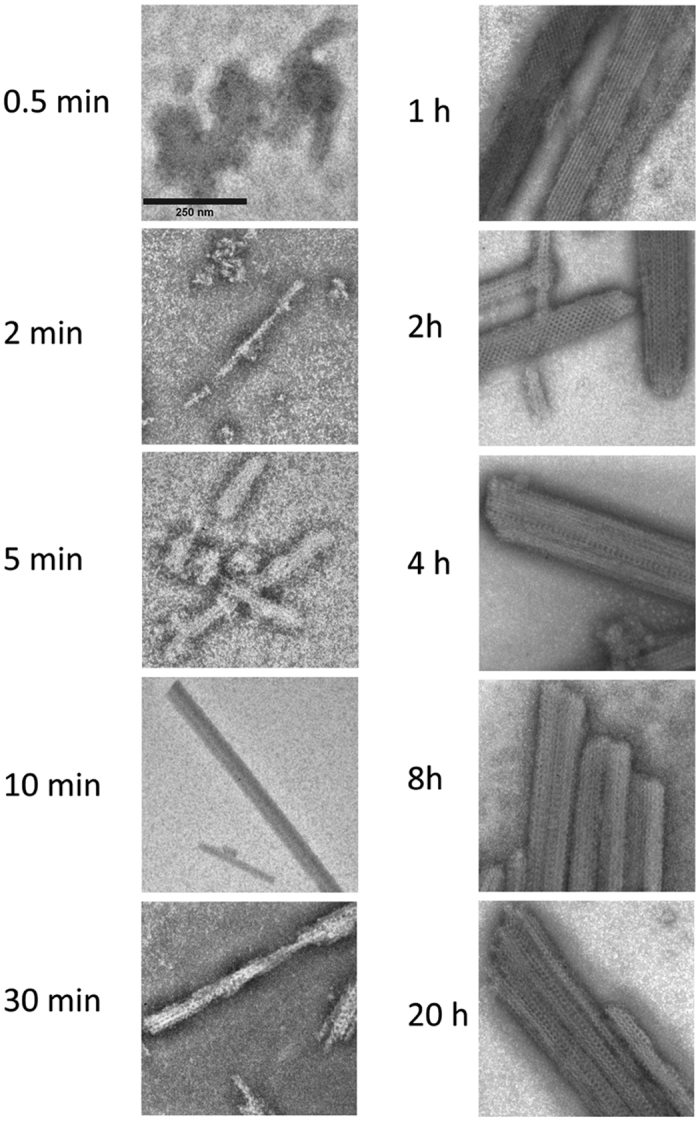
NS-TEM images of GalR^WT^ diluted in a buffer containing 250 mM NaCl and incubated for the indicated times (shown to the left) before aggregation was stopped by flash freezing the samples. The scale bar (top left) corresponds to 250 nm.

We used stopped-flow kinetics to study the rapid phase of aggregation upon dilution of GalR in a buffer containing lower [NaCl]. We observed simple kinetic traces (Fig. 3A), where a very short (~1 s) lag phase precedes a rapid increase in signal that gradually levels out, consistent with light scattering experiments. Data could be fitted to a double exponential decay (eq. 1) where the first phase represents the initial lag phase. We next investigated the structures formed at different stages of the aggregation process using NS-TEM (Fig. 4). In the rapid phase of aggregation (0–5 min), GalR condenses and forms initial aggregates with little organized structure. This is followed by a slower maturation phase (5–60 min), where highly ordered structures form. From here onwards, more ordered structures emerge which become longer and wider over the course of the next 8 h. When preformed aggregates were added to a solution of dimers and subjected to aggregating conditions, the aggregation process was not accelerated, indicating that formation of an aggregation nucleus was not a rate limiting step (Fig. S4A,B).

We also followed the development of aggregation over long time scales by light scattering. Samples incubated at different [NaCl] reached different end-point values after 8 h, although these values (Fig. 3B) were closer to each other than the plateau values reached after the first rapid phase (Fig. 1B). This implies that NaCl affected the initial phase more than the slow phase, and agrees with the observation that highly ordered structures are indeed able to form above 250 mM NaCl (depending on protein concentration, see previous section).

### Aggregate formation is accompanied by a change in the secondary structure

Far-UV CD spectra recorded during aggregation of GalR in 250 mM NaCl over 8 h revealed a transition in spectra (Fig. S5A) which we could model as a linear combination of the initial and final spectra, i.e. a simple A → B transition (Fig. 3C and S5A,B). Note that the first 10 min were not followed in this experiment (see *Methods*). The CD time course coincided well with overall changes in light scattering and the formation of ordered structures as visualized by NS-TEM (Fig. 4), implying that changes in secondary structure occurred as highly ordered structures emerged.

**Figure 5:**
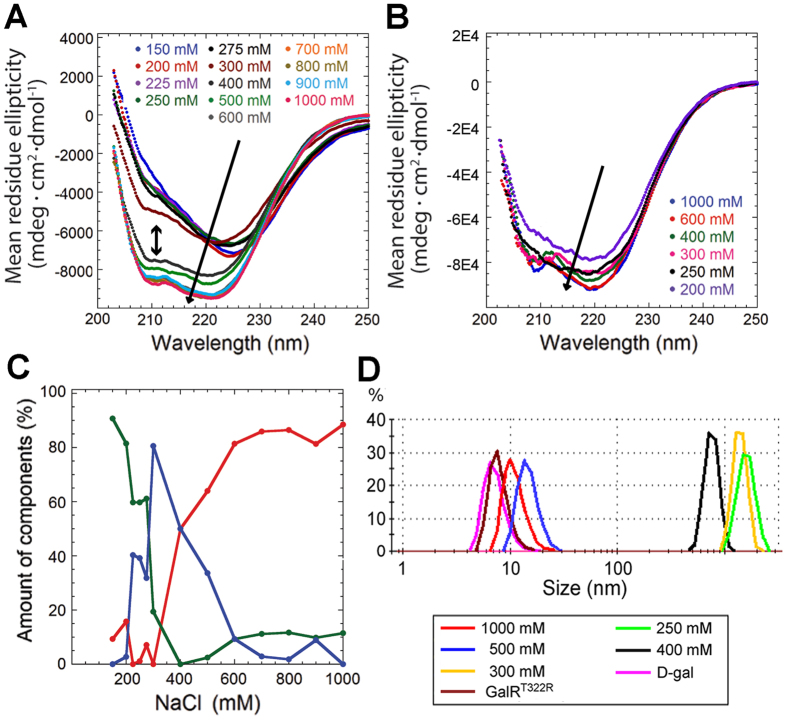
CD spectra of 3.8 μM GalR^WT^ (**A**) and GalR^T322R^ (**B**) diluted in buffers containing the indicated [NaCl] and incubated for 1 hour before the spectra were recorded. Arrows indicate increased [NaCl] from top to bottom, and the double-headed arrow in (**A**) indicates the separation of spectra below (top) and above (bottom) 400 mM [NaCl]. (**C**) Amount (% of total) of the three different CCA-obtained pure spectra plotted against [NaCl] giving rise to a three-stage model. (**D**) Number based size distribution of GalR^WT^ obtained using DLS at different [NaCl]. GalR^T322R^ was used with 250 mM NaCl. 10 mM D-galactose was used with 500 mM NaCl.

CD spectra of GalR recorded at different [NaCl] after 1 h of incubation showed a sharp transition between 400 and 300 mM NaCl (Fig. 5A). Above 400 mM NaCl, the spectra are dominated by an α-helical signal with distinct minima around 222 and 208 nm^17^. Below 300 mM NaCl, we observed a very different spectrum with a large minimum around 225 nm and a shoulder around 210 nm. The non-aggregating mutant GalR^T322R^ undergoes much smaller changes under these conditions (Fig. 5B). We used convex constraint analysis (CCA) to elucidate the minimum number of spectra (i.e. states) needed to describe the conformational shift of GalR during aggregation (see *Methods*). CCA takes as input the experimental CD spectra and the desired number of basis spectra. In addition to optimal basis spectra, CCA also yields the relative weights of each basis spectrum under all experimental conditions, which are indicative of the relative populations of each state^25^. There are two types of error that can cause experimental spectra to deviate from theoretical fits in CCA, namely an insufficient number of model states and experimental noise. We sought to identify the number of basis spectra at which fit improvement becomes noise-limited, after which addition of more basis spectra would simply overfit experimental noise. The semi-log plot of RMSD (root mean square deviation) versus numbers of spectra (Fig. S5C) shows a distinct kink when three spectra were used, indicating this to be a significantly better fit than with two spectra. Using four or more spectra resulted in only a modest fit improvement. To further elucidate the number of theoretical basis spectra that would provide a good fit to the experimental spectra without overfitting experimental error, we compared the RMSD of individual theoretical spectra with the corresponding experimental spectra as a function of [NaCl] (Fig. S5D). When two basis spectra were used, the RMSD revealed large errors at low and intermediate [NaCl]. When the number of basis spectra was increased to three (or higher), the errors were distributed approximately equally throughout the range of [NaCl], indicating that using three basis spectra provides a good fit to the experimental data without overfitting. Lastly, we also examined the relative populations of each state as a function of [NaCl] when three basis spectra are used to describe the experimental data (Fig. 5C). This plot shows that the relative populations of each state vary smoothly as a function of [NaCl] and that each of the three states predominates at different [NaCl]. In other words, all three states are necessary in order to properly describe the experimental data over the range of experimental conditions tested. The fall and rise of relative populations furthermore suggests a three-state sequential model (A → B → C). To investigate whether the observed three-state change could directly be coupled to aggregation, we monitored DLS on GalR samples under similar conditions (Fig. 5D). The data are consistent with the presence of 2–3 states: (i) A small (~7 nm diameter) state at high [NaCl], in the presence of *D*-gal or for the mutant GalR^T322R^. Based on previous studies^1^,^19^,^26^, this state likely represent a predominantly dimeric form. (ii) A slightly bigger (~15 nm in diameter), possibly tetrameric form at intermediate [NaCl]. (iii) Finally a very large state (aggregate, ~1 μm in diameter) at low [NaCl]. Note that the polydispersity index was generally high (~0.6), i.e. the solutions were very heterodisperse, where peaks at the lower end of the x-axis seem to be slightly asymmetric, tailing to larger sizes. This could result in the sizes mentioned above being slightly overestimated. Nevertheless, DLS and CD data are overall in general agreement.

### GalR aggregation is partially reversible

**Figure 6:**
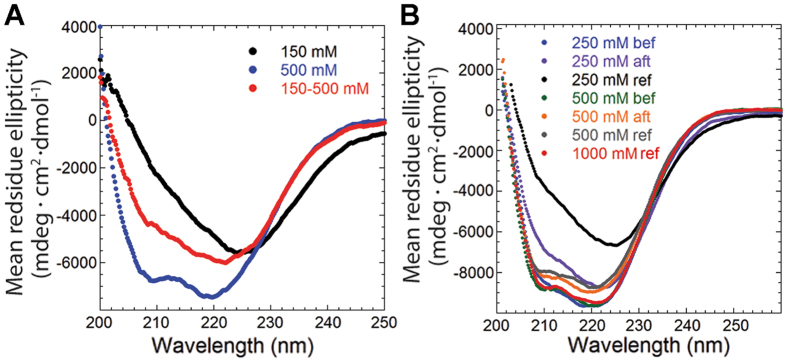
(**A**) CD spectra of 3.8 μM GalR diluted in buffers containing different [NaCl]. The label “150–500 mM” denotes an initial 1 h incubation with 150 mM NaCl after which the concentration was raised to 500 mM and incubated for an additional hour before this spectrum was recorded. (**B**) CD spectra of 3.8 μM GalR^WT^ at different [NaCl] and 10 mM *D*-galactose added either before (bef) or 1 hour after (aft) the protein was introduced. Spectra recorded in the absence of *D*-galactose at 250–1000 mM (ref) are included.

**Figure 7:**
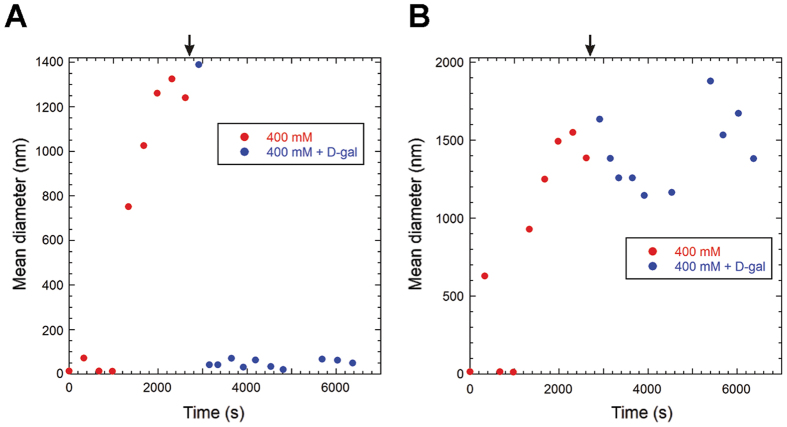
Mean sizes of GalR particles determined by DLS at different times after dilution of GalR in a buffer containing 400 mM NaCl. 10 mM D-galactose was added after the initial aggregation process had stabilized (arrow). The mean size calculation based on number PSD (**A**) gives equal weight to all particles, while in the calculation based on volume PSD (**B**) the weight of particles in the calculation is proportional to their GalR content.

We next investigated the disassembly of aggregates due to increased [NaCl]. GalR was allowed to aggregate at 150 mM NaCl for 1 h, after which the [NaCl] was raised to 500 mM and the sample was incubated for an additional 1 h. This led to partial reversion of the CD spectrum (Fig. 6A), indicating that aggregation is only partially reversible or that the conformational shift of GalR is a slow process. To further investigate the dynamics of the aggregates, we studied how GalR aggregates respond to the presence of *D*-gal (Fig. 6B). *D*-gal was able to prevent structural changes of GalR to a large extent, even at low [NaCl], but only if present from the beginning throughout the 2 hour incubation. *D*-gal could not fully disassemble the aggregated structures if GalR was preincubated in the low salt buffer for 1 h before adding *D*-gal. We therefore performed an experiment where GalR was diluted in a buffer containing a final [NaCl] of 400 mM (starting from 1000 mM NaCl) and followed the aggregation by DLS. At this [NaCl], light scattering showed the aggregation to be slow enough that the sample likely would not change significantly during the course of individual measurements. After the mean sizes had stabilized (~45 min), *D*-galactose was added to a final concentration of 10 mM (Fig. 7A,B, blue spheres). This resulted in the release of a large number of smaller species from the aggregates as the mean sizes from number derived particle size distribution (PSD) (Fig. 7A) strongly decreased. This is in agreement with previous studies^19^ where it was found that *D*-galactose prevents formation of, and likely dissociates, higher order oligomers. In contrast to this, mean sizes calculated based on the volume PSD (reflecting mean sizes weighted by particle volumes) indicated that considerable amount of large species were still present (Fig. 7B). This implies that aggregation is only partially reversible.

### Structural analysis reveals two classes of aggregates

**Figure 8:**
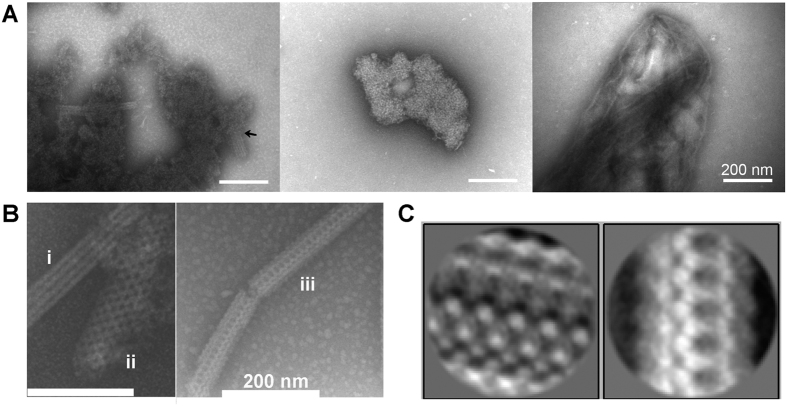
NS-TEM images of GalR aggregates formed at 3.8 μM protein and 250 mM NaCl. (**A**) Ordered structures which may act as seeds for the assembly of highly ordered structures, indicated by the arrowhead on the left panel. (**B**) Highly ordered striated structures (i), web-like structures (ii), and structures with prominent holes (iii). All images were taken at the same magnification. (**C**) Examples of 2D class averages showing the highly ordered substructure of the types of structures marked (ii) and (iii), left and right respectively.

From a visual inspection of 957 NS-TEM images, we were able to group the aggregates into two broad categories: Ordered structures and highly ordered structures. For the ordered structures, common traits include visible edges and sizes ranging from several hundred nanometers to above 1 μm. Apart from these shared features, the morphologies of the aggregates varied greatly (Fig. 8A), including both folded ribbons and condensed beads. We speculate that these structures may act as seeds for the assembly of highly ordered structures (Fig. 8A left). The highly ordered structures varied considerably in length (ca. 75–500 nm) and to a lesser extent in width (ca. 40–150 nm). However, they all shared three features: i) an overall rod-like appearance, ii) the presence of highly ordered substructures on the surface, and iii) association with less ordered aggregates (Fig. 1C). Three overall types of substructures were observed: (i) A striated appearance with a two-dimensional repeat structure, (ii) a web-like structure, and (iii) a structure with pronounced holes (Fig. 8B). Many of the highly ordered structures observed are a combination of two or more of the types described, suggesting considerable freedom in lattice formation or a lack of radial symmetry. Owing to their differences in width and length, the structures were not suitable for conventional single particle analysis. Instead, we attempted to generate high resolution reconstructions by averaging different sections of the individual structures, which were highly repetitive (Fig. 8C, with an example of sectioning shown in Fig. S6). One of the averaged structures, shown on the left in Fig. 8C, suggests an essentially spherical smallest subunit with a diameter of ~9 nm that is repeated in a tetragonal pattern. On the other hand, in the averaged structure shown on the right, a repeating hexagonal pattern is present, with the smallest subunit being less visible but still essentially spherical and with a diameter of ~8 nm. Both of these values lie close to the diameter measured for the dimer using DLS (~7 nm).

## Discussion

Even though GalR has long been known to aggregate at lower ionic strengths, this aspect of the protein has not received much attention until recently. Several lines of evidence suggest that the aggregation process has biological relevance. (i) Aggregates of GalR are observed *in vivo* in stationary phase cells^20^; (ii) Distant GalR binding sequences on the chromosome are spatially close to each other in cells when GalR is present; (iii) higher order multimers of GalR can bridge separate operator sequences *in vitro*^20^; and (iv) the transition from the dimeric to the polymeric form occurs at a physiologically relevant ionic strength.

In this study, we found that GalR aggregates can form highly ordered polymers with a fine substructure. Although the aggregates investigated here could easily be composed of thousands of GalR dimers and thus exceed realistic intracellular sizes, they could nevertheless still represent extended versions of the biologically relevant smaller aggregates.

The estimated *in vivo* concentration of GalR is 100–125 dimers per cell in log phase^20^,^27^, and may increase about 5-fold as cells enter stationary phase^28^. Over this entire concentration range, there is a robust linear correlation between GalR concentration and light scattering (Fig. S1C), suggesting that the GalR concentration is high enough to promote aggregation *in vivo*.

**Figure 9:**
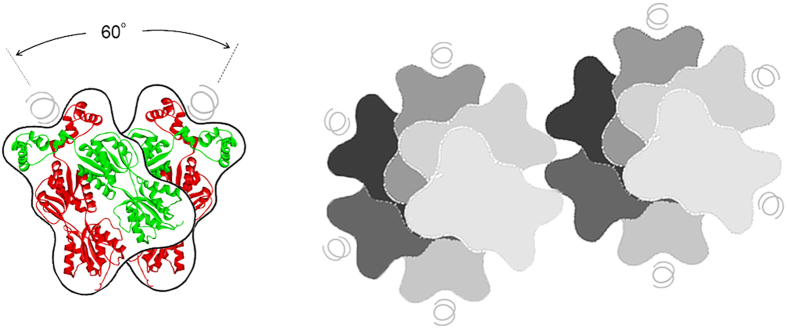
A schematic model of GalR polymerization. According to a structural proposal based on a combination of homology modelling and genetic data, GalR dimers form a V-shaped, stacked tetramer with a 60° twist angle (left)^11^. The tetramer contains free tetramerization interfaces. We propose that this will allow binding of further dimers, resulting in ‘linear’ oligomers. These oligomers can interact side by side using the N-terminal interface. The ‘cross section’ of the ‘linear’ oligomers and the predicted interaction of two oligomers is shown on the right. The DNA binding domains which are not hindered by the side by side interactions remain functional and allow binding of distant sites on the chromosomal DNA.

Assembly of the highly structured polymers requires a functional tetramerization interface. Hence, the mutant GalR^T322R^, which is impaired in tetramer formation, cannot form these structures. It is noteworthy that the homology structure of GalR suggests that tetramerization interfaces are found on both sides of the dimers^17^. This is further supported by the observation that the GalR^WT^/GalR^T322R^ heterodimer has only one functional tetramerization interface^29^. As a consequence, the tetramer composed of two stacked dimers has two available interfaces for further association of dimers^11^. This provides a way for the assembly of long strings of stacked dimers, which are likely required for the highly ordered structures to arise. A model representation of this is shown in Fig. 9. However, it is not clear how these long strings give rise to the larger structures. Our data indicate that the head pieces containing the helix-turn-helix DNA binding motif are involved in the assembly of the highly ordered structures. CD spectra suggest that aggregation involves two separate conformational changes, but these could also involve other parts of the protein and could occur subsequent to the initial assembly.

**Figure 10:**
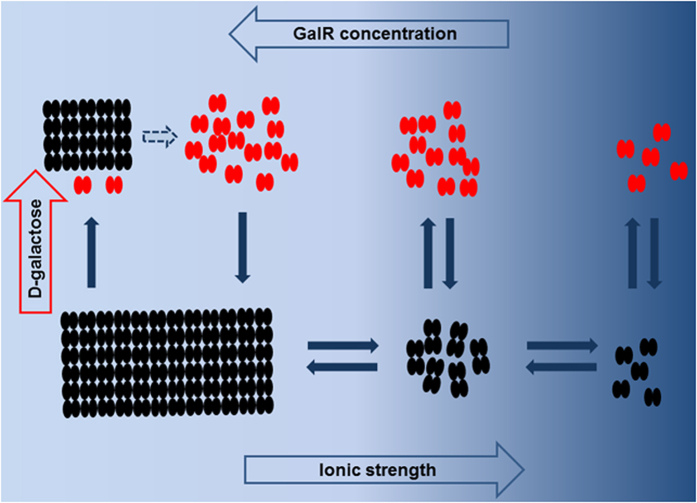
The model of GalR aggregation. At high ionic strength GalR (black ovals) is present predominantly in the dimeric form. At intermediate ionic strength dimers associate to oligomers in a GalR concentration dependent manner. At low ionic strengths GalR assembles to highly structured polymers. D-galactose inhibits GalR oligomerization and also disassembles oligomers, keeping GalR in the dimeric form (red). D-galactose also inhibits the assembly of the highly ordered polymers but preformed polymers are only partially disassembled.

Based on the above observations, we propose the following model for GalR aggregation (Fig. 10): Exposure to low ionic strengths directly induces a conformational shift which alters the tetramerization interface, allowing linear oligomerization of GalR dimers. Longer exposure allows a second conformational shift, which is induced by the association of the linear oligomers to form rods with highly ordered substructures. We speculate that the second conformational change affects folding of the DNA-binding domain for the following reasons: (i) the highly ordered structures do not form in the absence of the DNA-binding domain; (ii) the interaction of GalR dimers in these structures becomes more tolerant to the presence of *D*-galactose.

D-galactose binding induces large conformational changes in the GalR protein, affecting both DNA binding and tetramerization. A mutation which stabilizes the tetramerization interface^30^ or DNA binding to GalR^18^,^19^ both interfere with the galactose induced conformational shift. It has been shown that tetramerization of GalR in solution is more sensitive to *D*-galactose than DNA binding of the protein, *i.e.* the formation of the tetramerization interface is more galactose-sensitive than the folding of the DNA binding head piece^19^. Also, we assume that similar to the structurally analogous *Lac* Repressor, the hinge region connecting the DNA binding domain to the protein core is unfolded in the absence of DNA or in the presence of inducer^31^. Therefore, we suggest that the *D*-galactose tolerance of both the aggregates observed *in vitro* and *in vivo* results from the stabilization of the folded conformation of the DNA-binding head piece.

In conclusion, we propose that in the dynamic environment of growing cells, GalR functions as a galactose-responsive transcriptional regulator, while in stationary phase cells it can assemble to galactose-resistant tubules with well-defined substructural features which may assist chromosome architecture. The *in vitro* self-assembly process of these highly ordered tubules can be controlled in multiple ways, e.g. by GalR concentration, by salt concentration, by the presence of D-galactose or by the presence of assembly interface mutants, allowing regulation of self-assembly dynamics and size distribution. The DNA binding ability of the tubules provides a potential for the assembly of more complex structures.

## Methods

### Protein expression and purification

Phenotypes of GalR mutants used in this study are summarized in Table S1. GalR^WT^, GalR^Y244F^, GalR^T322R^, GalR^N48I^, and GalR^Δ46^ were expressed as hexahistidine-tagged fusion proteins under the control of a L-arabinose inducible promoter using the vector plasmid pSEM1026^12^. GalR was purified by a modified version of the method described previously^12^. The purification process resulted in 1 mg/ml (25.5 μM) GalR in a buffer containing 1 M NaCl; 50 mM Tris:Cl (pH 8); 10% (v/v) glycerol; and 5 mM 2-mercaptoethanol. The protein was aliquoted, flash-frozen in liquid nitrogen, and stored at −80 °C. Each experiment was performed with a new aliquot. GalR concentrations were determined from absorption at 280 nm using an extinction coefficient of 20500 M^−1^ cm^−1^(as predicted by the online tool ProtParam^32^) and a M_W_ of 39.2 kDa. For GalR^Δ46^ 19000 M^−1^ cm^−1^ and 34.4 kDa were used. Two batches of GalR^WT^ were purified and one batch of each mutant.

### Fluorescence

For aggregation studies, tryptophan fluorescence emission spectra were recorded on either a Varian Cary Eclipse fluorescence spectrophotometer (Agilent technologies, Santa Clara, USA) or a LS 55 fluorescence spectrometer (PerkinElmer, Waltham, USA). For each spectrum, 3 accumulations (150 nm/min) with excitation at 295 nm and emission at 310–450 nm were averaged.

### Light scattering (LS)

Light scattering experiments were performed on a Varian Cary Eclipse fluorescence spectrophotometer using excitation and emission wavelengths of 365 nm with bandwidth of 2.5 nm and stirring at room temperature. Aggregation was initiated by diluting a protein stock of GalR (25.5 μM; 1 M NaCl; 50 mMTrisCl pH 8; 10 (v/v)% glycerol; and 5 mM 2-mercaptoethanol) into reaction solutions with final concentrations: 2 μM GalR, 50 mM TrisCl pH 8 with NaCl in the 78–1000 mM range and Na_2_SO_4_ in the 25–700 mM range. Note that in the Na_2_SO_4_ experiments, 78 mM NaCl were present as well. Data was collected every 0.5 s over 20 min or every 5 s over 8 h with a recording time of 0.2 sec for each data point. Three aggregation experiments were performed using GalR^WT^ samples at 150 mM NaCl (two of which are shown Fig. 1A), and the results were very similar.

### Stopped-flow analysis

Stopped-flow experiments were carried out on a SX-18MV microreaction analyzer (Applied Photophysics, Leatherhead, UK). GalR was diluted by mixing 1:5 with buffer to a final concentration of 2 μM GalR and 150 mM NaCl. Fluorescence was recorded by excitation at 280 nm and a cut-off filter at 320 nm. Data was fitted to the equation


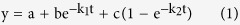


### Circular Dichroism (CD) spectroscopy

CD spectra were recorded on a JASCO J-810 spectropolarimeter (Jasco, Tokyo, Japan) in the 190–260 nm range at 100 nm/min scanning rate, a response time of 2 s, bandwidth of 2 nm, 1 mm solution path-length, and 3.8 μM (0.15 mg/ml) GalR with 5 accumulations. Individual background spectra were made for each NaCl and D-galactose concentration. Samples prepared at different [NaCl] (150–1000 mM) were preincubated for at least 45 min prior to data collection. For time-dependent data, a protein concentration of 2 μM and 1 ml final reaction volume in a 4 mm solution path-length quartz crystal cuvette at room temperature was used. One spectrum was collected every 10 min for 8 h in the 200–250 nm range with stirring throughout the experiment. The first spectrum was collected 10 minutes after the start of the aggregation process.

### Convex constraint analysis (CCA)

The convex constraint analysis was used to deconvolute CD spectra of GalR^WT^(13 in total), obtained at different [NaCl], into a number of pure component spectra. The algorithm yields a specified number of pure spectra, which, when properly weighted and summed, are approximations to all of the experimental spectra, all of which is performed by the algorithm. The analysis was performed using the CCA+ software^33^ with default settings and varying only the number of pure spectra. The validation of the results was inspired by^25^,^34^, comparing the quality of fits obtained performing CCA while varying the the number of pure component spectra between one and seven.

### Dynamic light scattering (DLS)

DLS data was collected using 6.4 μM GalR on a Zetasizer Nano ZS (Malvern, Worcestershire, UK), recorded at an angle of 173°. Samples were equilibrated at 20 °C for 2 min before data acquisition. The viscosity was set to that of water, and refractive index was set to standard protein. Using the Malvern software, attenuators and number of accumulations were automatically optimized for each measuring point. All experiments were repeated three times. Only median values are shown as the data was noisy and thus using the mean would give high weight to outliers.

### Negative staining transmission electron microscopy (NS-TEM)

GalR stock was diluted into 50 mM Tris:Cl pH 8, 3.8 μM (0.15 mg/ml) GalR, and 250 mM NaCl unless otherwise stated. At different time points, samples were flash-frozen in liquid nitrogen and prepared for negative contrast/staining as follows. Samples were thawed after which 5 μl sample was applied to the surface of a carbon-coated, glow discharged 400 mesh Ni grid. After 2 min, the grid was stained with 3 drops of 1% phosphotungstic acid (PTA) pH 7.0 and blotted dry on filter paper. Electron microscopy was performed using a JEOL 1010 TEM (JEOL (Germany) GmbH, Freising, Germany) at 60 kW. Images were taken using an Olympus KeenView (Olympus, Tokyo, Japan) camera. For size determination, a standard grid-size replica plate (2160 lines/mm) was used. For subsequent 2D classification and averaging individual super structures were chosen manually. Picking of boxes to be used as input in the 2D classification and averaging was done using the program e2boxer from the EMAN2 program package^35^. Three criteria were used when selecting the superstructures. i) The magnification was the same for all structures (25000X). ii) The structures were identified as single structures, *i.e*. not several structures lying next to each other iii) A clear boundary between background and structure. Boxes containing sections of the super structures were then loaded into the program packaged RELION^36^ where reference-free 2D classification was carried out with ten classes and 25 iterations using the default settings. No CTF correction was applied during image processing.

## Additional Information

**How to cite this article**: Agerschou, E. D. *et al* The transcriptional regulator GalR self-assembles to form highly regular tubular structures. *Sci. Rep.* **6**, 27672; doi: 10.1038/srep27672 (2016).

## Supplementary Material

Supplementary Information
