# Supplementary material for: The transcriptional regulator GalR self-assembles to form highly regular tubular structures
Source: Sci Rep. 2016 Jun 9;6:27672. doi: 10.1038/srep27672 (PMC4899725; doi:10.1038/srep27672)
Supplement: Supplementary Information [file srep27672-s1.pdf]

Supplementary information for

The transcriptional regulator GalR self-assembles to form highly regular tubular structures

by Emil D. Agerschou, Gunna Christiansen, Nicholas P. Schafer, Daniel Jhaf Madsen, Ditlev E. Brodersen, Szabolcs Semsey and Daniel Otzen

	DNA binding	D-galactose binding	Oligomerization	Polymerization	Ref
GalR^{WT}	+	+	+	+	1
GalR^{Y244F}	+	-	+	+	23
GalR^{T322R}	+	+	-	-	17
GalR^{N48I}	-		+	-	24
GalR$^{\Delta46}$	-		-	-	
GalR^{V7A}	-		+	+	24

Table S1. Phenotypes of GalR mutants. Red symbols indicate phenotypes found in this study.

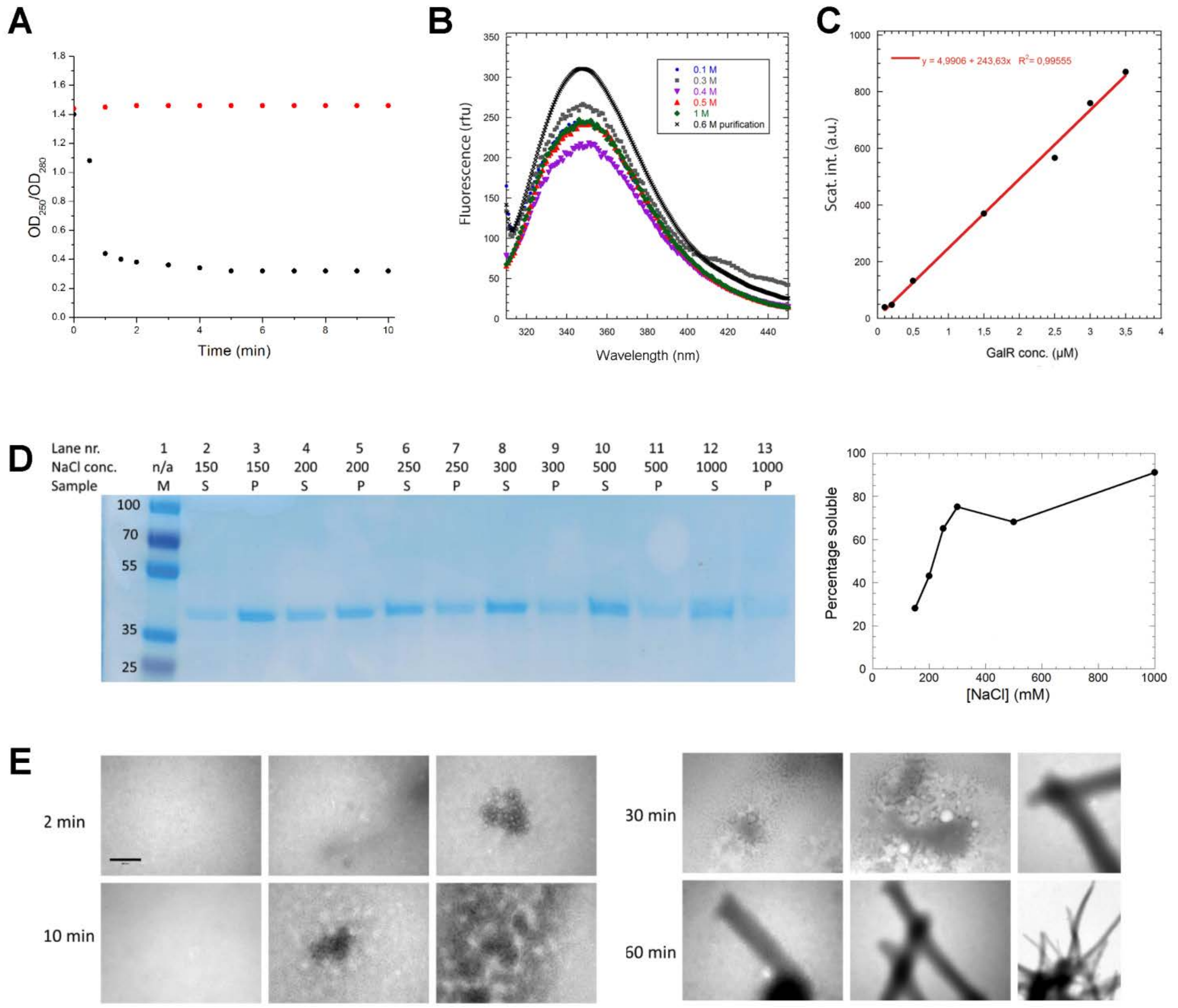

Figure S1. (A) Optical density after samples were preincubated in 100 mM NaCl for 1 h before addition of 1 mM D-Galactose to GalR^{WT} (black) and GalR^{Y244F} (red) with a final protein concentration of 0.1 mg/ml. Note that phosphate buffer was used. (B) Tryptophan fluorescence of GalR^{WT} in samples diluted to different [NaCl] after the light scattering plateau had been reached. A sample of GalR^{WT} purification using 0.6 M NaCl, resulting in aggregation, is included (0.6 M purification). (C) Light scattering intensity at plateau of GalR^{WT} diluted to different concentrations at a constant [NaCl] of 150 mM (black filled circles) and fit to a linear equation (solid red line). Similar results were obtained in the in vitro transcription buffer (17) containing 200 mM potassium glutamate. (D) SDS-PAGE of GalR^{WT} diluted to different [NaCl] where both the pellet (P) fractions and supernatant (S) fractions were loaded alongside a molecular maker (M) with size indicated to the right. The graph next to the gel shows the percentage of soluble protein at each [NaCl], based on densitometric analysis using ImageJ. (E) NS-TEM images of 0.4 mg/ml GalR^{WT} incubated at 400 mM NaCl for the time indicated to the left.

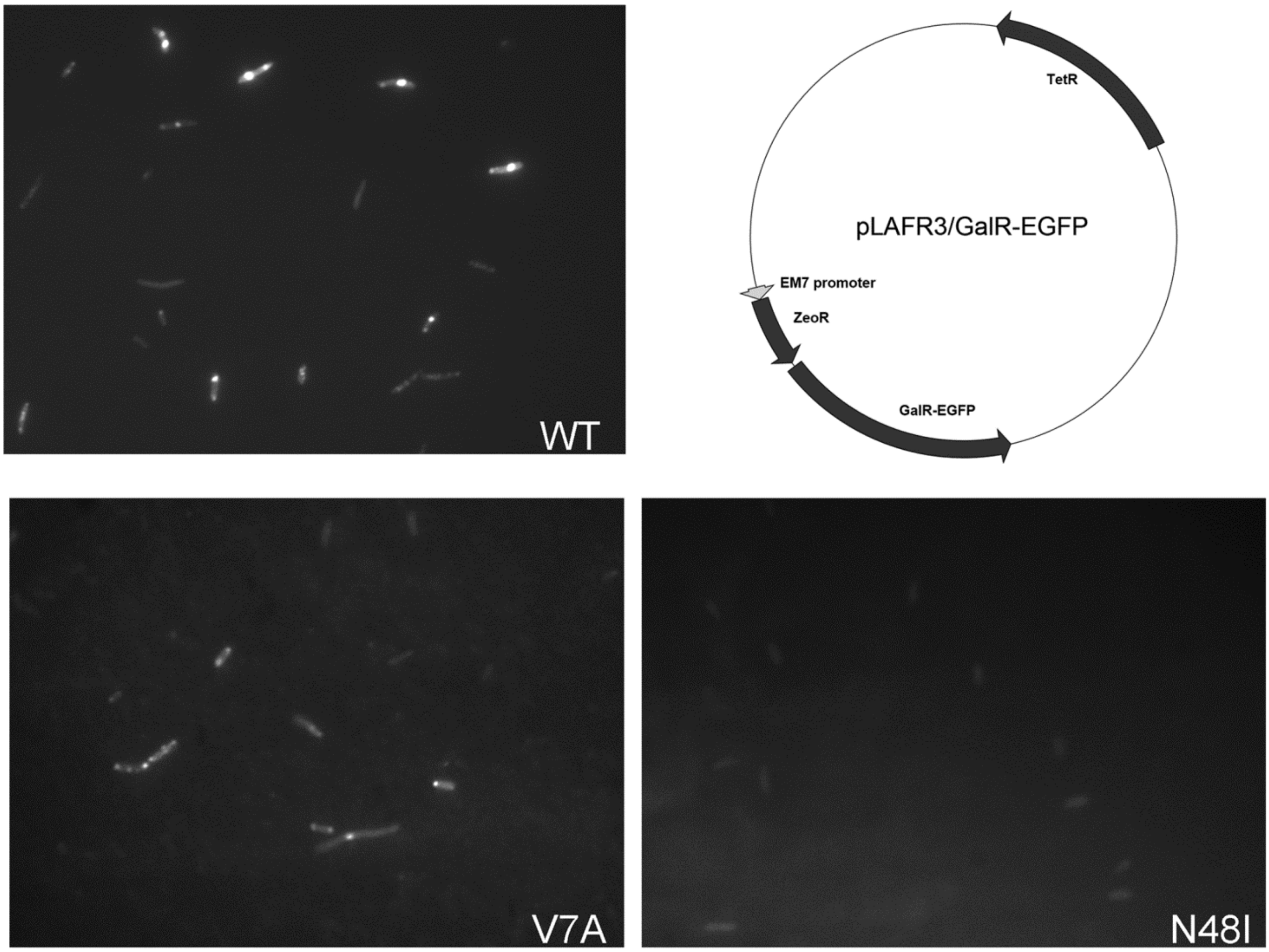

Figure S2. Fluorescence microscopic analysis of the distribution of EGFP labeled GalR in live stationary phase cells. In the pSEM1029 plasmid (12) the sequence encoding the EGFP protein was fused to the C-terminus of wild type GalR and of two non-binding mutants, V7A and N48I, which were identified in a previous study (24). The GalR-EGFP fusions, together with the EM7 promoter and the zeocin resistance marker, were inserted into the low copy plasmid pLAFR3 (Staskawicz, B.; Dahlbeck, D.; Keen, N.; Napoli, C., J Bacteriol 169:5789-94,1987). In the case of *E. coli* cells carrying the GalR^{V7A}-EGFP fusion, fluorescent foci could be observed, similar to cells carrying the GalR^{WT}-EGFP fusion. However, the GalR^{N48I}-EGFP protein did not form foci; it had a uniform distribution in stationary phase cells.

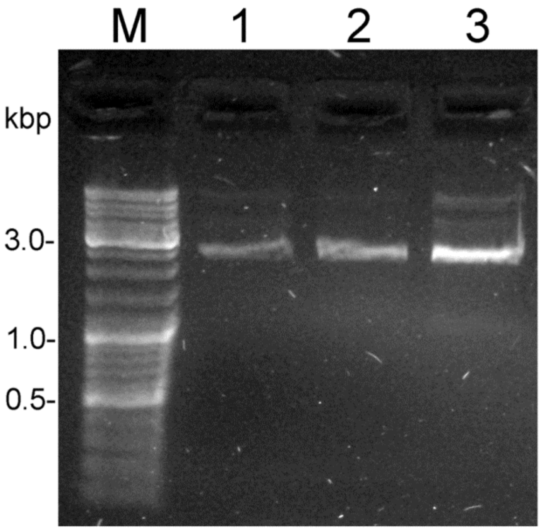

Figure S3. GalR aggregation in the presence of DNA. 10 µg GalR protein and 0.8 µg supercoiled pSA850 plasmid (10) DNA was incubated for 90 minutes in 200 µl buffer containing 10 mM Tris acetate at pH 7.8, 5 mM magnesium acetate, 100 mM potassium glutamate (and 50 mM NaCl from the GalR protein). The precipitate formed was centrifuged for 10 min at 15000 g. 20 µl of the supernatant (lane 1), the precipitate resuspended in 20 µl buffer (lane 3), and 80 ng of plasmid DNA in the same buffer without GalR (lane 2) were loaded on an agarose gel. The plasmid DNA was enriched in the precipitated fraction, suggesting that at least some of the subunits in the aggregates can bind to DNA.

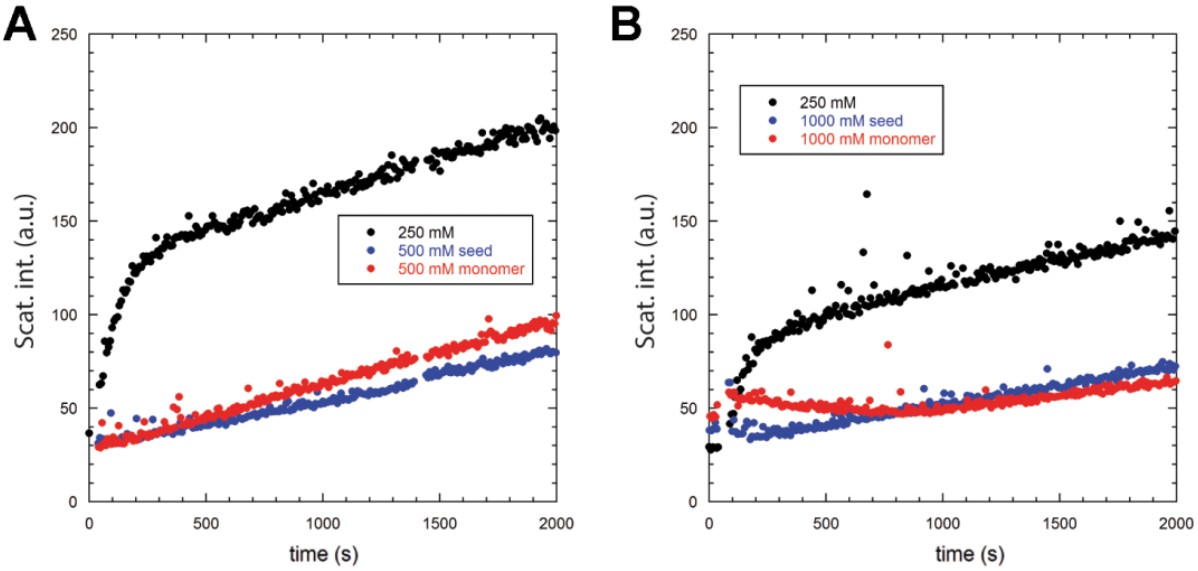

Figure S4. GalR^{WT} diluted to 250 mM NaCl and either 500 mM NaCl (A) or 1000 mM NaCl (B) and followed by light scattering as described in *Materials and Methods*. GalR^{WT} was aggregated by incubation at 250 mM NaCl. After 1000 s, aggregated GalR^{WT} ("250 mM") was diluted 100-fold into a solution with (A) 500 mM, and (B) 1000 mM NaCl and light scattering was followed for an additional 1000 s (giving the "500 mM seed" and "1000 mM seed" curves). In parallel, monomeric GalR was incubated at 500 and 1000 mM NaCl and the process followed over time ("500 mM monomer" and "1000 mM monomer").

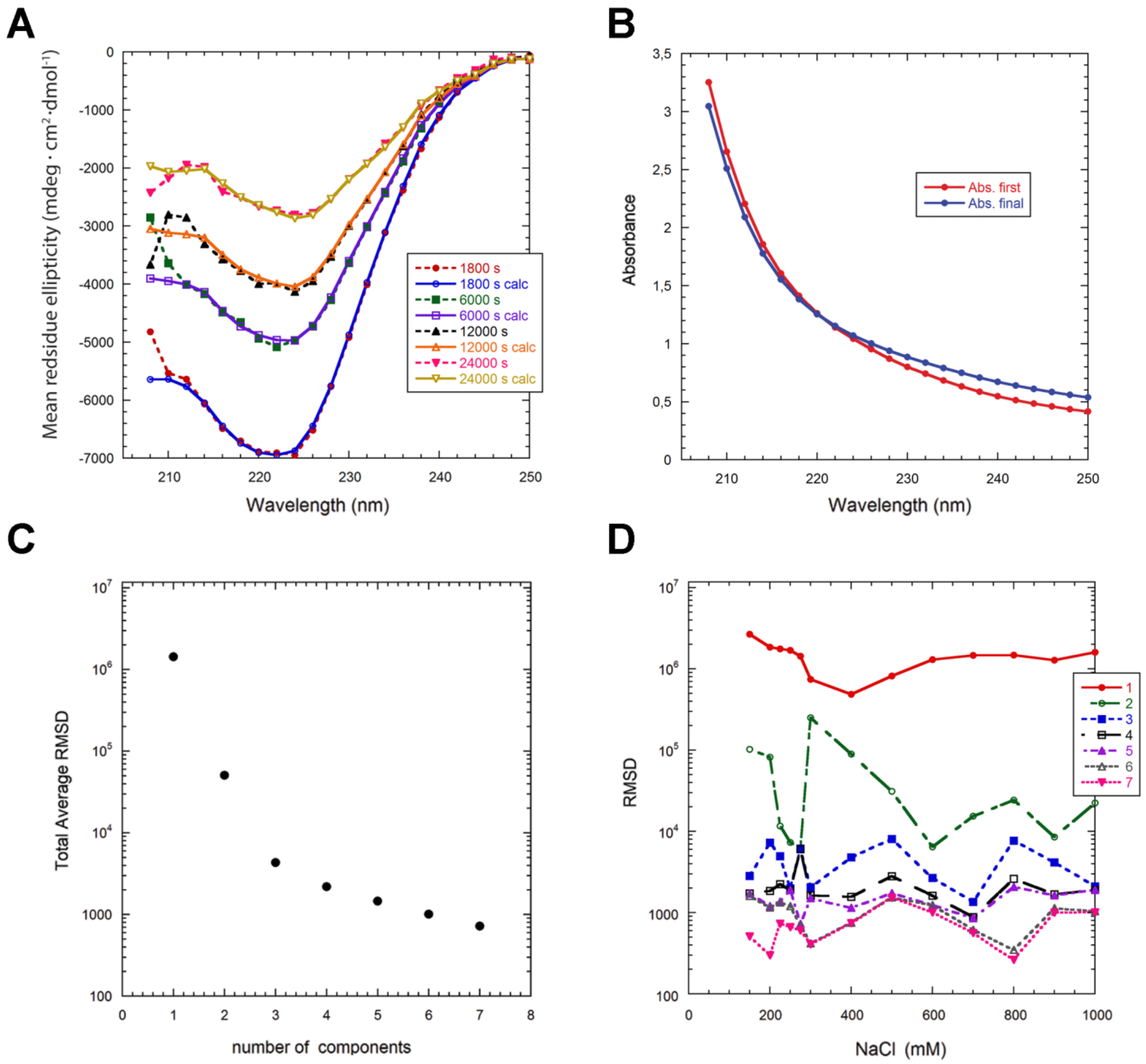

Figure S5. Changes in secondary structure accompanying aggregation of GalR. (A) Aggregation over time for 2 µM GalR in 250 mM NaCl followed by far-UV CD. Comparison between representative experimentally observed and calculated spectra (from a least square method, see main text) over the course of the 8 h CD experiment. (B) Absorbance over the entire wavelength spectrum of the first (10 min) and final (8 h) experimentally obtained CD spectra. Note that the spectrum was recorded in a 1 cm cuvette. CD spectra were recorded in 2 mm cuvettes, leading to a factor 5 lower absorbance, and thus allowing us to obtain reliable spectra down to 210 nm. (C) The total average RMSD plotted against the numbers of pure CCA spectra. Note that the y-axis is logarithmic. (D) RMSD plotted against [NaCl] for an increasing number of pure CCA structures.

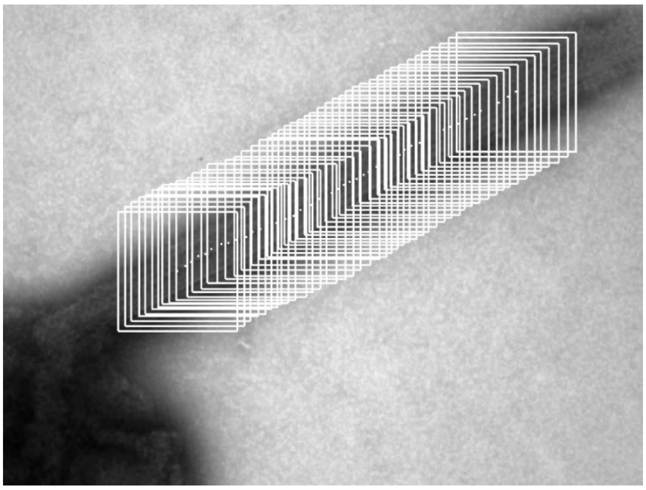

Figure S6. An example of sectioning (white boxes) of a highly ordered structure which was used as an input for the 2D-averaging of images of highly ordered aggregates.
